# Abiotic Stress-Responsive miRNA and Transcription Factor-Mediated Gene Regulatory Network in *Oryza sativa*: Construction and Structural Measure Study

**DOI:** 10.3389/fgene.2021.618089

**Published:** 2021-02-12

**Authors:** Rinku Sharma, Shashankaditya Upadhyay, Sudeepto Bhattacharya, Ashutosh Singh

**Affiliations:** ^1^Department of Life Sciences, Shiv Nadar University, Gautam Buddha Nagar, India; ^2^Department of Electrical Engineering, Indian Institute of Technology, New Delhi, India; ^3^Department of Mathematics, Shiv Nadar University, Gautam Buddha Nagar, India

**Keywords:** *Oryza sativa*, microRNA, transcription factor, regulatory network, post-transcriptional regulation, target mimics

## Abstract

Climate changes and environmental stresses have a consequential association with crop plant growth and yield, meaning it is necessary to cultivate crops that have tolerance toward the changing climate and environmental disturbances such as water stress, temperature fluctuation, and salt toxicity. Recent studies have shown that trans-acting regulatory elements, including microRNAs (miRNAs) and transcription factors (TFs), are emerging as promising tools for engineering naive improved crop varieties with tolerance for multiple environmental stresses and enhanced quality as well as yield. However, the interwoven complex regulatory function of TFs and miRNAs at transcriptional and post-transcriptional levels is unexplored in *Oryza sativa*. To this end, we have constructed a multiple abiotic stress responsive TF-miRNA-gene regulatory network for *O. sativa* using a transcriptome and degradome sequencing data meta-analysis approach. The theoretical network approach has shown the networks to be dense, scale-free, and small-world, which makes the network stable. They are also invariant to scale change where an efficient, quick transmission of biological signals occurs within the network on extrinsic hindrance. The analysis also deciphered the existence of communities (cluster of TF, miRNA, and genes) working together to help plants in acclimatizing to multiple stresses. It highlighted that genes, TFs, and miRNAs shared by multiple stress conditions that work as hubs or bottlenecks for signal propagation, for example, during the interaction between stress-responsive genes (TFs/miRNAs/other genes) and genes involved in floral development pathways under multiple environmental stresses. This study further highlights how the fine-tuning feedback mechanism works for balancing stress tolerance and how timely flowering enable crops to survive in adverse conditions. This study developed the abiotic stress-responsive regulatory network, APRegNet database (http://lms.snu.edu.in/APRegNet), which may help researchers studying the roles of miRNAs and TFs. Furthermore, it advances current understanding of multiple abiotic stress tolerance mechanisms.

## Introduction

Abiotic stresses such as drought, cold, and salt can reduce the productivity and yield of plants with a direct adverse impact on global food security (Myers et al., 2017). In the spontaneously changing climate scenarios of recent years, there has been an increase in episodes of occurrence and the severity of these stresses (Lesk et al., 2016). Rice (*Oryza sativa*) is the most imperative crop across the globe, grown in over a hundred countries (including India), with a production rate greater than 700 million tons per annum (Londo et al., 2006). Researchers have estimated that around 1% of enhancement per annum in the *O. sativa* yield is required to fulfill increasing population demands (Normile, 2008). The literature on this subject includes several studies on rice, examining ways to enhance its nutritional quality and tolerance toward many diseases (Grover and Minhas, 2000). Increasing the yield of rice to meet this increasing demand is one of the most challenging aspects of this research, as various abiotic stresses adversely affect production (Mantri et al., 2012).

Plants have developed dynamic responses at the morphological, physiological, and biochemical levels that allow them to escape and/or adapt to calamitous environmental conditions. Plants regulate these responses at the molecular level through a series of complex interwoven network of events. Transcription factors (TFs) and microRNAs play an important role in regulating the activity of the genes at transcriptional and post-transcriptional levels, respectively, involving a complex series of events (Hobert, 2008; Li and Zhang, 2016; O’Brien et al., 2018).

Transcription factors belong to multi-gene families and have DNA-binding and protein-protein interaction domains through which they interact with cis-elements of their target genes and oligomerize with other TFs or with other regulators, respectively (Boeva, 2016). They aid in the regulatory system in several ways, by managing stress-responsive gene expression at the correct time and place, and controlling developmental and defense responses (Kissoudis et al., 2014; Shao et al., 2015; Samad et al., 2017). A single TF can control the expression of several genes in a particular pathway. Furthermore, recent studies have firmly linked the expression of a gene to the expression of TFs. For example, miR169 at the mRNA level controls the expression of the NFYA5 TF. It was shown in a transgenic plant experiment that suppression of *NFYA5* gene expression leads to susceptibility toward drought stress in plants (Li et al., 2008). This proves them as potential targets for the manipulation of desired traits in plants.

MicroRNAs (miRNA) are a major class of small endogenous RNAs of length 20–24 nucleotides. They assemble with ARGONAUTE (AGO) proteins forming an RNA-induced silencing complex (RISC) in the cytoplasm. AGO protein has PAZ and PIWI domains. PIWI domain creates an RNaseH-like fold that helps in cleaving RNA targets complementary to the miRNA strand assembled with the AGO in RIS-complex. It is often found that miRNAs regulate the target genes at the protein level without causing major change at the mRNA level. These findings suggest the capability of plant miRNAs to control gene expression at mRNA and protein levels (Voinnet, 2009; O’Brien et al., 2018). Recent studies have revealed the role of miRNAs in attenuating plant growth and development under the influence of several environmental stresses. Induction of miRNA expression under stress leads to repression of target genes, whereas, their repression leads to the expression of target genes under stressful conditions. The miRNAs play a central role in complex gene regulatory networks and are studied as a novel target for plant improvement, including improved tolerance to various environmental stresses. For example, over-expression of miR156 enhances tolerance to heat stress in *Arabidopsis thaliana* (Stief et al., 2014) and increases biomass in switchgrass (Fu et al., 2012). Over-expression of miR402 brings more tolerance to salinity, drought, and cold stress in *A. thaliana* (Kim et al., 2010). TFs and miRNAs play an essential role in multiple stress conditions. Recent studies have revealed that several TFs and miRNAs show similar expression patterns in response to multiple stresses (Zhang, 2015; Wang et al., 2016). This indicates that they could be targets for multiple abiotic stress-tolerant variety development.

In the literature on this subject, many miRNAs, TFs, and mRNAs were reported in response to multiple stress conditions in different plant species using computational programs and deep-sequencing techniques. Researchers have also studied the potential role of miRNAs, and TFs in gene expression control through several experimental methods. Over the past few years, however, there has been a shift in interest toward deciphering the complex interwoven regulatory networks operating in plants. The studies conducted either describe transcriptional or post-transcriptional regulation of genes, but a comprehensive study is lacking (Chen et al., 2018; Sun and Dinneny, 2018; Haque et al., 2019). Several online plant resources also exist for searching transcriptional or post-transcriptional regulation of genes. These include- (a) PlantRegMap (Jin et al., 2017), which contains information about TFs and its direct target genes for 135 plant species integrated from literature mining, TF ChIP-seq, and prediction combined TF binding motifs and regulatory elements; (b) AtRegNet (Palaniswamy et al., 2006), which provides information about TFs and the direct target genes of *A. thaliana* integrated from experimental methods like- EMSA, ChIP-seq, yeast one-hybrid analysis, etc.; and, (c) AtmiRNet contains information about the transcriptional regulation of miRNAs based on experimental methods like—EMSA, yeast one-hybrid analysis, transgenic plant expressing an inducible TF-GR (glucocorticoid receptor) fusion protein experiments, etc. PASmiR and miRNEST are comprehensive literature curated databases for stress-responsive miRNA and its targets (Zhang et al., 2013; Szczesśniak and Makabowska, 2014). However, no database or repository provides a global view of transcriptional and post-transcriptional integrated regulation of gene expression under abiotic stress. To acknowledge this current issue, the present study constructed abiotic stress-responsive gene regulatory networks for *O. sativa* and studied them in-depth using the network theoretic approach. We developed a comprehensive database, APRegNet database,^1^ whose construction is discussed for abiotic responsive gene regulatory networks at both transcriptional and post-transcriptional level for *A. thaliana*, *O. sativa*, and *Zea mays* in response to drought, cold, salt, and waterlogging stress. This database provides a valuable resource for contemporary researchers.

## Materials and Methods

### Data Sources

We retrieved the small RNA and mRNA (GPL2025) expression high-throughput datasets of cold, drought, and salt stress conditions (Supplementary Table 1) from the public domain, GEO (Gene Expression Omnibus^2^) and ArrayExpress Archive.^3^

The *O. sativa* AGO1-associated sRNA HTS (high-throughput sequencing) datasets GSM455962, GSM455963, and GSM455964 were downloaded from the GEO database (Supplementary Table 1).

The *O. sativa* degradome sequencing datasets – GSE17398 and GSE19050 (Supplementary Table 1) were downloaded from the GEO database.

The miRNA sequences were downloaded from miRBase (release 21^4^) (Kozomara and Griffiths-Jones, 2014). We retrieved the transcripts of *O. sativa* genes and the gene annotations from the *O. sativa* Genome Annotation Project (RGAP), available at ftp://ftp.plantbiology.msu.edu/pub/data/Eukaryotic_Projects/o_sativa/annotation_dbs/pseudomolecules/version_7.0/all.dir/ (Kawahara et al., 2013).

### Finding Dysregulated Genes and miRNAs Related to Abiotic Stress

#### Differentially Expressed Genes Identified by Transcriptome Meta-Analysis

The GCRMA R package (Wu et al., 2004) was used to normalize the raw expression data and outlier samples were detected by the ArrayQualityMetrics R package (Kauffmann et al., 2009). Thereafter, transcriptome meta-analysis was performed for differentially expressed genes (DEGs) identified using function RPadvance in the Bioconductor package (Hong et al., 2006) and pathway analysis using the KEGG database (Kanehisa et al., 2017).

#### Differentially Expressed miRNAs Identification

The HTS raw reads were pre-processed, which comprise adaptor trimming, low-quality tags removal, and determining sequence quality check. We also summarized clean tag length distribution and common and specific sequences between samples. The sequences of rRNA, scRNA, snRNA, tRNA, exon, intron, and repeat sequence tags were removed using GenBank^5^ and Rfam (12.2) database.^6^ The cleaned reads were then aligned to reference genome (MSU7) RGAP, available at ftp://ftp.plantbiology.msu.edu/pub/data/Eukaryotic_Projects/o_sativa/annotation_dbs/pseudomolecules/version_7.0/all.dir/. The sequence coordinates were then compared to *O. sativa* miRNA GFF file and different measures of the expression level were generated, such as, the read count (total number of reads assigned to the reference RNA), adjusted read count (read count normalized by the number of times that the read maps to the library or the genome) and normalized RPM (reads per million) which was done by miRanalyzer (Hackenberg et al., 2009). The EdgeR and DeSeq R package were utilized for differential expression analysis of miRNA of drought, cold and salt stress conditions.

### AGO1-Enrichment Analysis of Differentially Expressed miRNA

The differentially expressed miRNAs from various abiotic stresses were subjected to AGO1-enrichment analysis by applying the following rules: (1) the miRNA should be detectable in at least one of the AGO1-associated sRNA HTS datasets and (2) its normalized accumulation levels should be three RPM or higher.

### Abiotic Stress Responsive TF-miRNA Induced Gene Regulatory Network Construction and Network Measure Calculation

The regulatory network covered five types of regulatory relationships: TF-gene, TF-miRNA, TF-TF, miRNA-gene, and target_mimics-miRNAs. We extracted the TF regulatory information from EGRINs (Environmental gene regulatory influence networks) for *O. sativa* (Wilkins et al., 2016). The miRNA regulatory activity is also controlled by a kind of ribo-regulator known as target_mimics. A 23-nucleotide sequence conserved in plant species in a family of non-coding RNAs resembles a cleavable miRNA target site, however, the site is not cleaved and instead negatively regulates miRNA activity through mimicry (Franco-Zorrilla et al., 2007). The target_mimics regulatory information were obtained from PeTMbase (plant endogenous target mimics database) database (Karakülah et al., 2016) and miRNA regulation of genes was predicted by the psRNATarget tool (Dai and Zhao, 2011) using default parameters and we validated the predicted targets through degradome sequencing data analysis, which was performed by CleaveLand v4.4.4 (Addo-Quaye et al., 2009).

We then examined certain local and global properties, namely; the scale-free behavior (Clauset et al., 2009), small-world-ness, assortative mixing, strongly connected components, and network centralities (degree, closeness, and betweenness) according to methods outlined in previous studies (Upadhyay et al., 2017; Sharma et al., 2020).

### APRegNet: Database Construction

#### Data Sources

**FIGURE 1:**
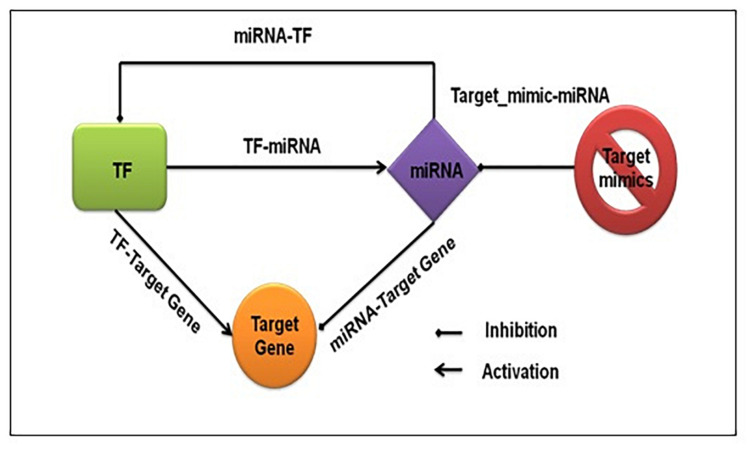
Fundamental regulatory interactions at the transcriptional and post-transcriptional level. The transcription factor regulating the expression of functional genes, other transcription factors, and miRNA, and at the post-transcriptional level miRNA regulating the expression of functional genes and transcription factors. The target mimics regulates miRNA expression.

We captured both transcriptional and post-transcriptional regulation of genes under abiotic stress in *A. thaliana*, *O. sativa*, and *Z. mays*. We considered TFs, miRNAs, and target_mimics as regulators. Figure 1 shows the fundamental regulatory interactions among – TF, miRNA, target gene, and target_mimics and also the essential steps of transcriptional and post-transcriptional regulation of gene expression.

We performed transcriptome meta-analysis for DEGs and miRNAs identification [for *O. sativa* discussed in this manuscript, for *A. thaliana* (Sharma et al., 2020) and *Z. mays* (unpublished)]. Thereafter we fetched the transcriptional regulation of genes, miRNAs, and other transcription factors and post-transcriptional regulation of miRNA by target_mimics from reliable databases restricted to experimentally validated interactions only (Table 1). We validated the miRNA regulation of target gene expression through degradome sequence meta-analysis [for *O. sativa* discussed in this manuscript, for *A. thaliana* (Sharma et al., 2020) and *Z. mays* (unpublished)].

**TABLE 1:** Data source: for abiotic stress (cold, drought, salt, and waterlogging) responsive genes and miRNAs regulatory transcription factors relationships extracted from various databases.

**Source**	**Description**	**Species**	**Link**
ATRM: *Arabidopsis thaliana* transcriptional regulatory map	ATRM database is a curated a high-confidence *Arabidopsis thaliana* transcriptional regulatory map derived by a systematic literature mining.	*Arabidopsis thaliana*	http://atrm.cbi.pku.edu.cn/
AtmiRNET: resconstructing regulatory networks of *Arabidopsis thaliana*	AtmiRNET database contain manually curated information about transcriptional regulation of *Arabidopsis thaliana* miRNAs derived from literature	*Arabidopsis thaliana*	http://atmirnet.itps.ncku.edu.tw/home.php
PlantRegMap: plant transcriptional regulatory map	PlantRegMap database contains genome-wide transcriptional regulatory interactions curated from literature and inferred by combining TF binding motifs and regulatory elements.	132 plant species	http://plantregmap.gao-lab.org/
Environmental gene regulatory influence networks (EGRINs)	This research article provided information about gene regulation network for *Oryza sativa* in response to high temperatures, water deficit, and agricultural field conditions by systematically integrating time-series transcriptome data, patterns of nucleosome-free chromatin, and the occurrence of known cis-regulatory elements.	*Oryza sativa*	PMCID: PMC5134975, 10.1105/tpc.16.00158
ArrayExpress	Contain gene expression data from Array and sequencing techniques	–	https://www.ebi.ac.uk/arrayexpress/
PlantTFDB (plant transcription factor database)	Contain highly curated information about 320,370 transcription factors from 165 plant species	Plants	http://planttfdb.cbi.pku.edu.cn/index.php
mirBase	An archive of miRNA sequences and annotation	–	http://www.mirbase.org/
NCBI GEO (Gene Expression Omnibus)	Contain gene expression data from Array and sequencing techniques	–	https://www.ncbi.nlm.nih.gov/geo/
TAIR (The *Arabidopsis thaliana* Information Resource)	TAIR database provides genetic and molecular biology data for *Arabidopsis thaliana*	*Arabidopsis thaliana*	https://www.arabidopsis.org/
*Oryza sativa* Genome Annotation Project	Contain genome sequence from the Nipponbare subspecies of *Oryza sativa* and annotation of the 12 *Oryza sativa* chromosomes.	*Oryza sativa*	http://rice.plantbiology.msu.edu/
Gramene-*Zea mays*	A curated, open-source, integrated data resource for *Zea mays*	*Zea mays*	http://ensembl.gramene.org/Zea_mays/Info/Index
PeTMbase	A database of plant endogenous target mimics (eTMs)	Plants	http://petmbase.org

KEGG pathway,^7^ PlantTFDB,^8^ INTERPRO,^9^ and DAVID^10^ sources were also integrated for Supplementary Information.

#### Database Implementation and Web User Interface Design

We developed an apprehensible and user-friendly web interface, APRegNet (see text footnote 1) for users to query and download the regulatory relationships and networks. APRegNet focuses on providing better navigation through individual sections to increase data discoverability. There are five tabs provided at the top of the interface (“Home,” “About,” “Source,” “Browser,” “Download,” and “Contact Us”) through which users can navigate and explore the required information. It runs on a XAMPP web server with an MYSQL database in the backend for data storage and management. Text query box is provided at the top of each page to search by various types of components (i.e., by TF, miRNA, or gene in the regulatory networks), by stress (cold, drought, salt, and waterlogging), and by species (*A. thaliana*, *O. sativa*, and *Z. mays*). The query result page shows results in three sections:

(1) **Network properties:** The network properties (in-degree, out-degree, closeness, and betweenness) of the queried component (gene/TF/miRNA), confined to selected species and stress-specific regulatory network;
(2) **Functional annotation:** This section includes Gene Model, Primary gene Symbol, GO term: Biological process, GO term: Molecular Function, GO term: Cellular Component, Transcription factor family, and KEGG pathway.
(3) **Interaction:** Displays first interacting patterns of the query component and type of interaction between source and target.

**FIGURE 2:**
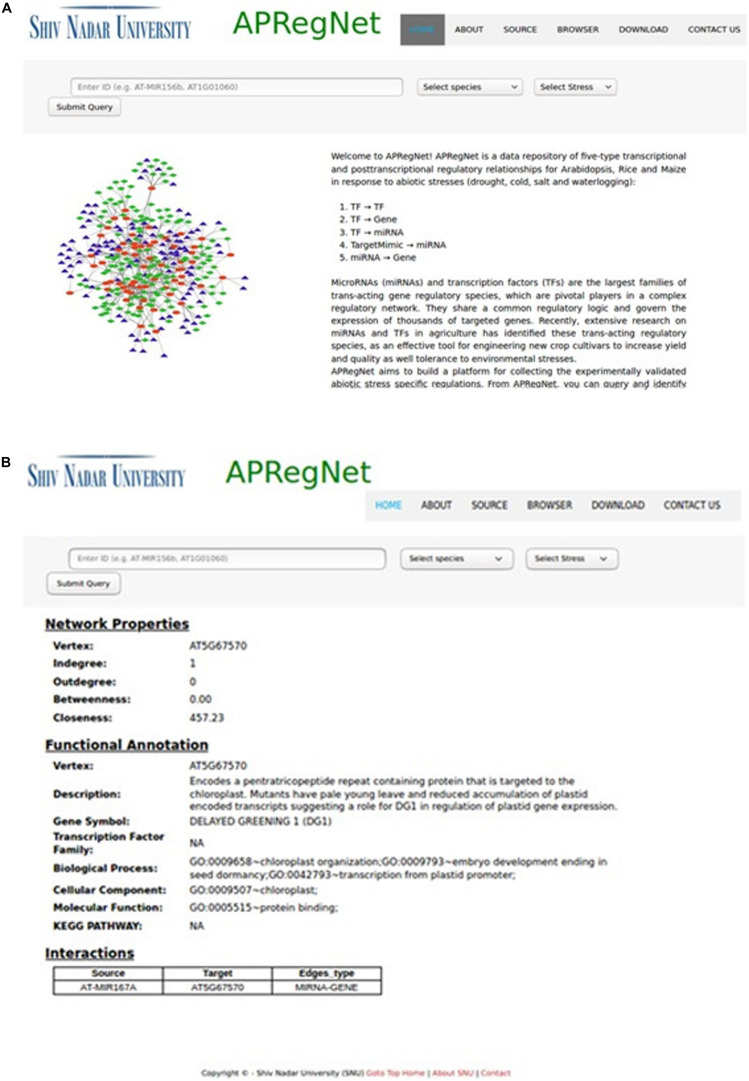
HomePage and result page of APRegNet Database. **(A)** Depicts the interface of the home page of APRegNet where the user can search information by typing the relevant keywords in the search tab and **(B)** shows the search result page.

The user can download the complete regulatory network in CSV file format for each species stress-wise from the download page. Figure 2 illustrates the snapshots of the database.

**FIGURE 3:**
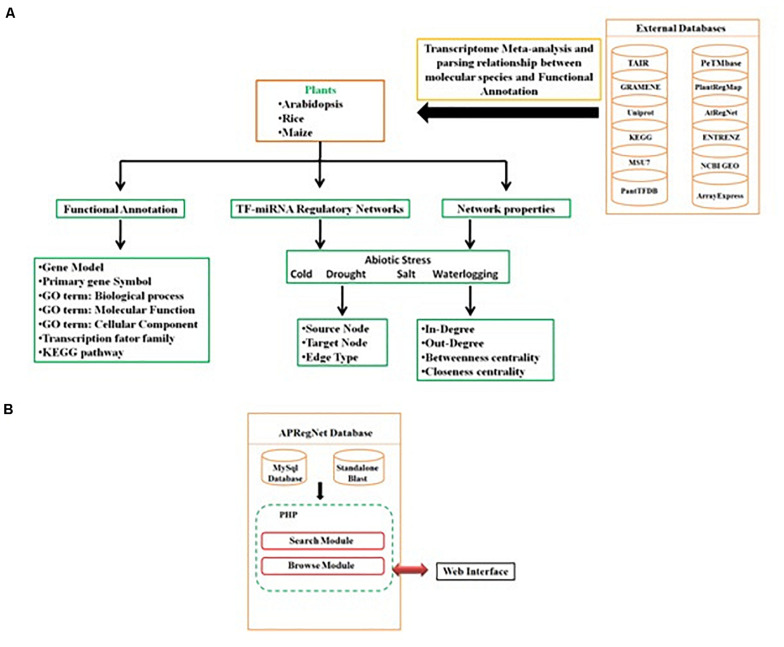
Schema of APRegNet Database. **(A,B)** Explains the method and data source for regulatory network generation and arrangement of various files in the database.

Query processing scripts are written in PHP and SQL. The database is tested and works well with commonly available web browsers, such as Mozilla Firefox, Google Chrome, Safari, and Microsoft Internet Explorer. The schema of the database is given in Figure 3.

## Results

In this study, miRNAs and transcription factors were woven into a complex inter-regulatory network within the cell liable for reprogramming gene expression in response to abiotic stresses in *O. sativa* deciphered through transcriptome (both coding and non-coding) meta-analysis. We further looked into the structural perspective of the proposed abiotic responsive networks which elucidated the significant role of some genes/TF/miRNA in the abiotic stress response mechanism.

### Abiotic Stress-Responsive Genes Identification by Meta-Analysis

**FIGURE 4:**
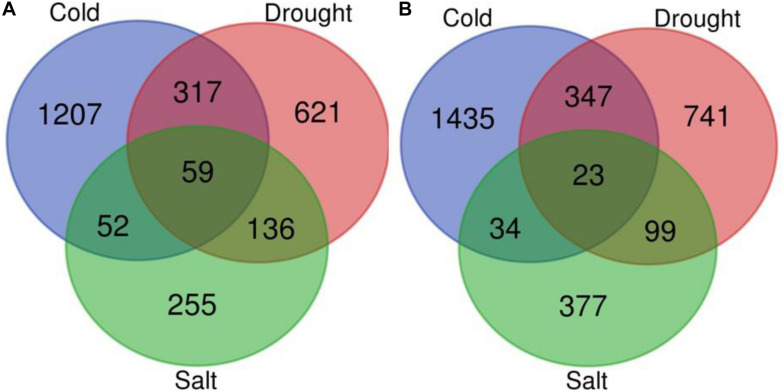
Venn diagram for differentially expressed genes. Venn diagram depicting the number of up **(A)** and down-regulated **(B)** genes under drought, cold, and salt stress in *Oryza sativa*.

We performed a meta-analysis of 15 studies of gene expression in response to abiotic stresses (cold, drought, and salt stress) (Supplementary Table 1) for each of the 51,279 genes using the RankProd method. The meta-analysis of 15 individual transcriptome profiling studies identified 5,255 genes showing significant differential expression in response to at least one of the abiotic stresses under investigation compared to control conditions in *O. sativa* [PFP (percentage of false positives) <0.01; Figures 4A,B, Supplementary Figure 2A, and Supplementary Table 2]. The comparative study of DEGs across the three stresses showed that 201 genes were commonly expressed across the three stresses among which 82 genes have conserved expression patterns. By contrast, a stress pair-wise comparison of the DEG lists found that drought and cold stress share the maximum number of DEGs, and over 75% of them showed similar expression patterns under both stresses. Functional annotation showed that among the conserved genes group, carbohydrate, nitrogen, and chlorophyll metabolism-related genes are under-expressed but that universal stress response proteins, hormonal signal transduction pathway, transport, energy production, and conservation-related genes were over-expressed across all the three stresses. The results showed that 73% (3,859) of the total DEGs were stress-specific (Figures 4A,B, Supplementary Figure 2A, and Supplementary Table 2).

### Abiotic Stress-Responsive miRNAs Identification

**FIGURE 5:**
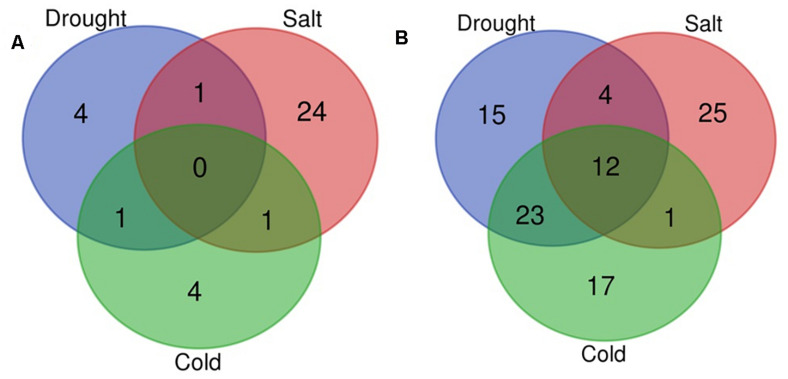
Venn diagram for differentially expressed miRNAs. Venn diagram depicting the number of up **(A)** and down-regulated **(B)** miRNAs under drought, cold, and salt stress in *Oryza sativa*.

In the present study, with the aid of publically available abiotic stress-specific sRNA HTS data for *O. sativa*; we identified drought, cold, and salt stress-responsive miRNAs. Supplementary Table 3 shows the number of miRNAs identified in control and each stress sample. Differential expression analysis also showed that 129 miRNAs were differentially expressed in response to at least one abiotic stress under study (Supplementary Table 4, Figures 5A,B, and Supplementary Figure 2B). A comparative study revealed that in response to salt stress, the highest numbers of miRNAs differentially expressed (68: 26 up-regulated and 42 down-regulated), followed by drought stress (60: 6 up-regulated and 54 down-regulated), and then cold stress (59: 6 up-regulated and 53 down-regulated). We found that under all three stresses, 12 DE miRNAs were common, having the same expression direction and stress-pair-wise comparison, which revealed that drought and cold stress shared maximum DE miRNAs (36) with conserved expression pattern followed by drought and salt stress (19) and then cold and salt stress (16). We observed that common miRNAs also have conserved expression patterns across the stresses. Several miRNAs showed differential expression unique to the abiotic stress category.

### Argonaute 1 Enrichment Analysis

The miRNAs, at the post-transcriptional level, regulate the target gene expression by target cleavage. They escort RISCs to the target mRNA for degradation or translational repression. A recent study has reported that ARGONAUTE protein (AGO1) selectively binds with miRNAs and short interfering RNAs in plants and catalyzes the cleavage of target mRNAs (Baumberger and Baulcombe, 2005; Arribas-Hernández et al., 2016). The present study identified AGO1-enriched miRNAs and found 91 AGO1-enriched miRNAs in response to abiotic stresses for *O. sativa* (Supplementary Table 5).

### AGO1-Enriched miRNAs Target Genes Identification

The targeted genes for abiotic stress-responsive AGO1-enriched miRNAs were initially predicted using psRNAtarget, a web-based online search tool under default parameters. As a result, 15,698 target binding sites predicted for 91 AGO1-enriched miRNAs expressed under abiotic stresses (Supplementary Table 6). These predicted target genes for miRNAs were validated through the meta-analysis of *O. sativa* degradome sequencing datasets, which is a high-throughput technique for large-scale validation of the miRNA-target duplex interactions (German et al., 2009). In the present analysis, six degradome sequencing datasets of *O. sativa* were downloaded from the public domain and used for performing a comprehensive validation of the predicted gene targets of abiotic stress-responsive miRNAs using CleaveLand4 version 4.4 pipeline (Addo-Quaye et al., 2009). It plots the sequenced tag abundance on each transcript and further grouped the cleaved target transcripts into five categories based on the relative abundance of the degradome tags mapping at the miRNA target site through the height of the degradome peak at each occupied transcript position (Categories 0, 1, 2, 3, and 4) (Supplementary Table 7).

A total of 538 psRNAtarget predicted (AGO1-enriched) miRNA-target interaction was validated by degradome sequencing data analysis from which 63 were classified as Category 0, 2 as Category 11, 82 as Category 2, 9 as Category 3, and 373 as Category 4. Category 0: >1 raw tags at the position, abundance at the position was equal to the maximum on the transcript. There was only one maximum on the transcript; Category 1: >1 raw tag at the position, abundance at the position was equal to the maximum on the transcript. There was over one maximum position on the transcript; Category 2: >1 raw tag at the position, abundance at the position was less than maximum but higher than the median for the transcript; Category 3: >1 raw tag at the position, abundance at the position was equal to or less than the median for transcript; Category 4: only 1 raw tag at the position. The validated interactions involved 445 genes and 80 miRNAs highly abundant under the influence of abiotic stresses (Supplementary Table 8).

### Abiotic Stress-Specific TF-miRNA-Gene Network Construction

We constructed the abiotic stress-responsive miRNA-TF gene regulatory networks for *O. sativa* in response to drought, cold, and salt stress. The networks contain five types of regulatory information: miRNA regulating genes, TF regulating genes, TF regulating TF, TF regulating miRNA genes, and target-mimics regulate miRNAs. The transcription factor regulation of abiotic responsive genes and miRNAs were derived from EGRINs, target-mimics regulation of abiotic stress-responsive miRNAs was derived from the publically available database: PeTMbase. The abiotic stress-responsive miRNAs validated targeted genes through degradome sequence data analysis. After assembly of all these relations, we built large directed graphs comprising (1352, 13546), (1063, 9901), and (949, 5920) nodes-edges pairs showing the relationship between genes, miRNA, and transcription factors in response to cold, drought, and salt stress, respectively. To study the nature of the constructed networks (a random or complex or regular type of network), the degree distribution was studied in the context of the power law using the method described by Clauset et al. (2009). The parameters that qualify the in-degree and out-degree sequences for the networks shown as a Supplementary Table 11. We found that each network in-degree and out-degree distribution follows power law as shown by the straight line in log-log plots, which is a distinctive nature of a complex network with non-random degree distribution, possibly a scale-free network (Supplementary Figure 1). Further, assortativity of all the networks computed and results displayed negative values of assortative mixing for in-in, out-out, in-out, and out-in degree pairs across the networks that revealed the interaction between nodes having the higher degree with nodes having a lower degree (Table 2). This showed the disassortative nature of all the present study networks which revealed the importance of hub nodes, i.e., nodes with the highest connectivity or degree, in case of failure of these hub nodes the network is likely to become disconnected which leads to no flow of information in the system and thus the system is disrupted.

**TABLE 2:** Topological attributes of the *Oryza sativa* abiotic stress-responsive TF-miRNA-gene networks.

Topological attributes	Rice		
	Cold	Drought	Salt
Number of edges	13,546	9,901	5,920
Number of nodes	1,352	1,063	949
Largest SCC size	42	43	43
Graph diameter	12	12	12
Characteristic path length	3.708	3.704	3.718
The average number of neighbors	18.993	18.087	11.926
Assortativity (in-in)	−0.523	−0.529	−0.457
Assortativity (in-out)	0.013	0.017	0.014
Assortativity (out-in)	−0.305	−0.249	0.017
Assortativity (out-out)	−0.470	−0.473	−0.437

We examined whether the networks had small-world properties or not. We computed transitivity (T_*G*_) and average shortest path length (ASL_*G*_) for three TF-miRNA induced stress-specific networks and compared their values with transitivity T_*ER*_ and average shortest path length ASL_*ER*_ of the Erdos–Renyi random network of the same order and sizes. We then calculated small-world-ness (S) for cold, drought, and salt stress-specific regulatory networks. The results showed that all the networks had a value >1, which suggested the small-world nature of all three networks (Table 3).

**TABLE 3:** Calculations of small-world-ness of the *Oryza sativa* miRNA-TF-gene regulatory networks.

Network	*Oryza sativa*				
	Transitivity_	Transitivity_	ASD_Net	ASD_Rand	Sw-ness
	Net	Rand			
Drought	0.027	0.017	2.842	2.716	1.533
Salt	0.042	0.012	3.329	3.011	3.118
Cold	0.022	0.014	3.020	2.755	1.403

Various network centrality measures calculations prioritized the molecular species (miRNA/TF/gene) of the networks. The details of the top 10 nodes of each stress-specific network of O. *sativa* are given in Supplementary Table 9.

In the directed graph like- regulatory networks of the present study, the two nodes “x” and “y” belong to the same strongly connected component, if there are directed paths both from “x” and “y” and from “y” and “x” (Jeong and Berman, 2008). We found that one SCC is present in drought, cold, and salt stress-specific regulatory networks. Supplementary Table 10 contains details of the TFs present in SCC. Functional analysis revealed the role of the SCC components in multiple abiotic stress responses, hormonal signal transduction, flower development, and cell fate specification.

### APRegNet Database

The APRegNet database developed by integrating the experimentally validated regulatory interactions between TFs, miRNAs, genes, and target_mimics from various sources; as a comprehensive repository for genome-wide regulatory networks operating in *A. thaliana*, *O. sativa*, and *Z. mays* in response to abiotic stresses (cold, drought, salt, and waterlogging). It contains regulatory relationships at both transcriptional and post-transcriptional levels as well as interaction among TF/miRNAs and their targets with easily downloadable options. It also provides the data source information for the regulatory interactions. Table 4 lists the basic statistics of the regulatory networks in APRegNet. This database contains regulatory information for 4,063, 2,026, and 7,152 genes/TFs/miRNAs for *A. thaliana*, *O. sativa*, and *Z. mays*, respectively.

**TABLE 4:** Basic statistics of the database: number of nodes, edges, TF (transcription factors), miRNAs (microRNA), stress-responsive genes, and various relationships among them (C, cold stress; D, drought stress; S, salt stress; and W, waterlogging stress).

	*Arabidopsis thaliana*				*Oryza sativa*			*Zea mays*			
Elements	D	C	S	W	D	C	S	D	C	S	W
Nodes	1,971	2,058	1,865	251	1,063	1,352	949	1,129	3,182	2,523	2,760
Edges	3,807	3,720	3,787	401	9,901	13,546	5,920	9,247	24,829	16,955	21,287
TF	369	354	393	163	340	390	337	307	378	386	464
miRNA	24	31	45	12	34	30	52	56	29	100	41
Gene	1,578	1,673	1,427	76	689	932	560	766	2,775	2,037	2,255
TF→TF	410	386	432	195	1,454	1,450	1,428	2,105	2,045	2,113	2,049
TF→Gene	3,251	3,134	3,091	133	8,299	11,917	4,098	6,148	22,279	13,172	18,521
TF→miRNA	76	91	128	40	3	38	30	431	231	678	288
TargetMimic→miRNA	22	45	47	9	16	5	28	151	97	283	137
miRNA→Gene	48	64	89	24	129	136	336	412	177	709	292

The query result page displays few network centrality measures (namely, degree centrality, closeness centrality, and betweenness centrality) of the query component (gene/miRNA/TF) related to specific species stress network, which quantifies the importance of the component in the network for the quick and efficient flow of information.

## Discussion

### *Oryza sativa* Abiotic Stress Responsive miRNA-TF-Gene Regulatory Networks

Cells use signal transduction pathways and regulatory mechanisms to coordinate multiple processes, allowing them to respond to and adapt to an ever-changing environment. The biological system of components that interact with or regulate each other can be represented by a mathematical object called a graph (Bollobás and Cockayne, 1979), which comprises nodes and edges. Since, in a biological system, the flow of information among the components is directional; the edges are therefore directed. The understanding of the features that emerge from the entire cellular function requires an integrated, theoretical description of the relationships between different cellular components. The *O. sativa* abiotic stress-responsive miRNA and transcriptional factor gene regulatory networks in the present study were constructed and quantitatively described using the network theoretical approach. The observed topologies of the regulatory networks provide clues about the influence of the organization on the function and dynamic responses of the plant toward multiple abiotic stresses.

A study of the structural measures of the constructed regulatory networks revealed the scale-free and small-world nature of the abiotic stress-responsive miRNA-TF-gene regulatory networks. This implies that the networks are highly tolerant to random failures within the system, a quick and efficient flow of environmental stress signal from the source to sink takes place, which helps the plant adjust to a stressful environment (Barabási and Albert, 1999; Newman, 2003; Humphries and Gurney, 2008; Ghoshal and Barabási, 2011; Sharma et al., 2020). The networks are stable and invulnerable to scale change, and even the removal of a significant number of non-hub nodes (TF/miRNA/gene) cannot affect global behavior, but continuity in signal flow may be affected if hub nodes are inactivated. As in small-world networks, the fraction of non-hub nodes is larger than hub nodes, meaning that instances of hub node failure barely happen (Albert et al., 2000).

The analysis of centrality measures showed various MIKC_MADS transcription factor family members as the best-ranked nodes under all three stress (namely, cold, drought, and salt) responsive regulatory networks in *O. sativa* (Supplementary Table 9). This TF family member plays a crucial role in flowering time, floral organ identity determination, and fruit ripening (Theissen and Melzer, 2007; Li et al., 2016). According to the ABCDE flower development model, A, B, C, D, and E are different classes of genes, and interaction in a specific combination of these gene classes specifies different floral organs- Class A + E genes specify sepals, A + B + E specify petals, B + C + E specify stamens, C + E specify carpels, and C + D + E specify ovules (Zahn et al., 2006; Silva et al., 2016). According to centrality measures, in each of the three stress-responsive networks, these four classes of genes were among the top-ranked nodes. For example, Class A genes: OsMADS14, OsMADS15, and OsMADS18; Class B gene: OsMADS2; Class C genes: OsMADS3 and OsMADS58; and Class E gene: OsMADS6 (Supplementary Table 9). Recent studies have shown that these classes of genes are important not only in plant growth and development but also in connection with abiotic stress responses in *O. sativa*, wheat, and brachypodium (Arora et al., 2007; Wei et al., 2014; Ma et al., 2017). When the interacting partners of these genes were explored in the networks under study, we found that they interact with other TFs and stress-responsive genes (such as NAC, HSF, SNF1, WRKY, bHLH, PP2C, chaperones, etc.) having a significant role in multiple abiotic stress. Further SCC analysis also revealed that the MIKC_MADS TF family genes are present, along with other abiotic stress-responsive TFs in the strongly connected component of the networks under all three stresses (Supplementary Table 10). This shows they may play a significant role in managing timely floral development under multiple stress conditions in *O. sativa*.

### APRegNet Database

Contemporary investigation of literature about transcription factors and miRNAs mediated regulation associated with plant abiotic stresses indicate that transcription factors and miRNAs act as key regulators in plant physiological adaption mechanisms during the response to various stress conditions. Information regarding the transcriptional and post-transcriptional regulation by TFs and miRNAs during plant responses to abiotic stress is distributed over numerous recent studies. The APRegNet database (see text footnote 1) construction provides a comprehensive repository of abiotic stress-responsive transcriptional and post-transcriptional regulatory networks.

This database contains knowledge-based abiotic stress-specific regulatory networks in *A. thaliana*, *O. sativa*, and *Z. mays*, and was developed by incorporating various data sources. It is a comprehensive collection of the interactions among TFs, miRNAs, and genes, occurring in response to abiotic stress, reconstructed for public access. The established regulatory networks from APRegNet provide genome-wide regulatory interactions that lay an initial foundation and establish a prior background network to identify or verify molecular and functional regulations in pathways.

Within a plant species under multiple abiotic stresses, certain miRNAs or transcription factors show common expression. The study of such miRNAs and TFs using a systematic, comprehensive database approach like APRegNet (see text footnote 1), will expedite the enhancement of understanding of the stress-specific transcriptional and post-transcriptional regulatory role of transcription factors and miRNAs and their functional evolutionarily relationship in various plant species. The regulatory information stored in this database has promising uses for experimental biologists who intend to improve plant crop performance under multiple Abiotic stress environments.

We also include additional information such as links to other databases (Uniprot, TAIR, MSU7, NCBI Gene database, and MaizeGDB). We are planning to extend the APRegNet to include other plant species and capture information about more abiotic stress responses.

## Conclusion

A complex interwoven network of multiple molecular species at various steps such as- transcription, post-transcription, translation, and post-translation control the expression of a gene. The alteration in expression of the gene in response to environmental cues enables the plant to acclimatize and survive in adverse environments, but this complex interwoven regulatory network is mostly unrevealed. In the present study, abiotic stress-responsive miRNA-TF-gene regulatory networks for *O. sativa* were reconstructed and analyzed to reveal information about how environmental stress induces these regulatory networks. Network structural measures were studied using the network theoretical approach, and deciphered several important features of the networks such as- scale-free, and small-world behavior that established the stable nature and quick, efficient transmission of signals (environmental cue). This process also highlighted the genes, TFs, and miRNAs shared by multiple stresses, working as a hub or bottleneck for signal propagation.

In crop plants, the right flowering time is pivotal for acclimatizing under the altered environmental condition and directly linked to grain yield (Gao et al., 2014). Under stress conditions, if flowering occurs prematurely, then seed set and grain-filling are adversely affected. If flowering is delayed, then there is the chance that plants may die without producing seeds. This study showed the interaction between stress-responsive genes (TFs/miRNAs/other genes) and genes involved in floral development pathways. However, this opens up further questions about how the fine-tuning feedback mechanism works in balancing stress tolerance and timely flowering to survive adverse conditions.

In summary, this study postulated some structural features of the miRNA and transcription factor regulated networks operating in a cell under the influence of multiple environmental stresses and prioritize the mainstream function of miRNAs and transcription factors in gene expression control.

## Data Availability Statement

The original contributions presented in the study are included in the article/Supplementary Material, further inquiries can be directed to the corresponding author/s.

## Author Contributions

RS, SU, SB, and AS conceived and designed the study. RS and SU performed the analysis. RS wrote the manuscript. All authors read and approved the manuscript. All authors contributed to the article and approved the submitted version.

## Conflict of Interest

The authors declare that the research was conducted in the absence of any commercial or financial relationships that could be construed as a potential conflict of interest.

## Footnotes

1 http://lms.snu.edu.in/APRegNet

2 http://www.ncbi.nlm.nih.gov/geo/

3 https://www.ebi.ac.uk/arrayexpress/

4 http://www.mirbase.org/

5 http://www.ncbi.nlm.nih.gov/GenBank/

6 http://rfam.sanger.ac.uk/

7 https://www.genome.jp/kegg/pathway.html

8 http://planttfdb.cbi.pku.edu.cn/

9 https://www.ebi.ac.uk/interpro/

10 https://david.ncifcrf.gov/

## Supplementary Material

Supplementary Figure 1 Degree distribution plot

Supplementary Figure 2 Venn diagram for up and down regulated differentially expressed **(A)** mRNA and **(B)** miRNAs

Supplementary Table 1 Details of transcriptome raw data.

Supplementary Table 2 List of differentially expressed genes under cold, drought, and salt stress in *Oryza sativa*.

Supplementary Table 3 miRNA-seq data analysis result.

Supplementary Table 4 List of differentially expressed miRNAs under cold, drought, and salt in *Oryza sativa*.

Supplementary Table 5 AGO1-enriched miRNA differentially expressed under abiotic stresses.

Supplementary Table 6 List of miRNAs targets predicted by psRNATarget.

Supplementary Table 7 List of miRNAs targets identified by degradome sequencing data analysis.

Supplementary Table 8 List of miRNAs targets validated by degradome sequencing data analysis results.

Supplementary Table 9 Top 10 genes/TF/miRNA ranked by degree, betweenness, and closeness centrality in *Oryza sativa* abiotic stress-induced regulatory networks.

Supplementary Table 10 Details of SCC found in three *Oryza sativa* abiotic stress-responsive miRNA-TF-gene regulatory networks.

Supplementary Table 11 Scale free network analysis.
