# Supplementary material for: Abiotic Stress-Responsive miRNA and Transcription Factor-Mediated Gene Regulatory Network in Oryza sativa: Construction and Structural Measure Study
Source: Front Genet. 2021 Feb 12;12:618089. doi: 10.3389/fgene.2021.618089 (PMC7907651; doi:10.3389/fgene.2021.618089)
Supplement: Supplementary Figure 1 — Degree distribution plot [file Data_Sheet_1.PDF]

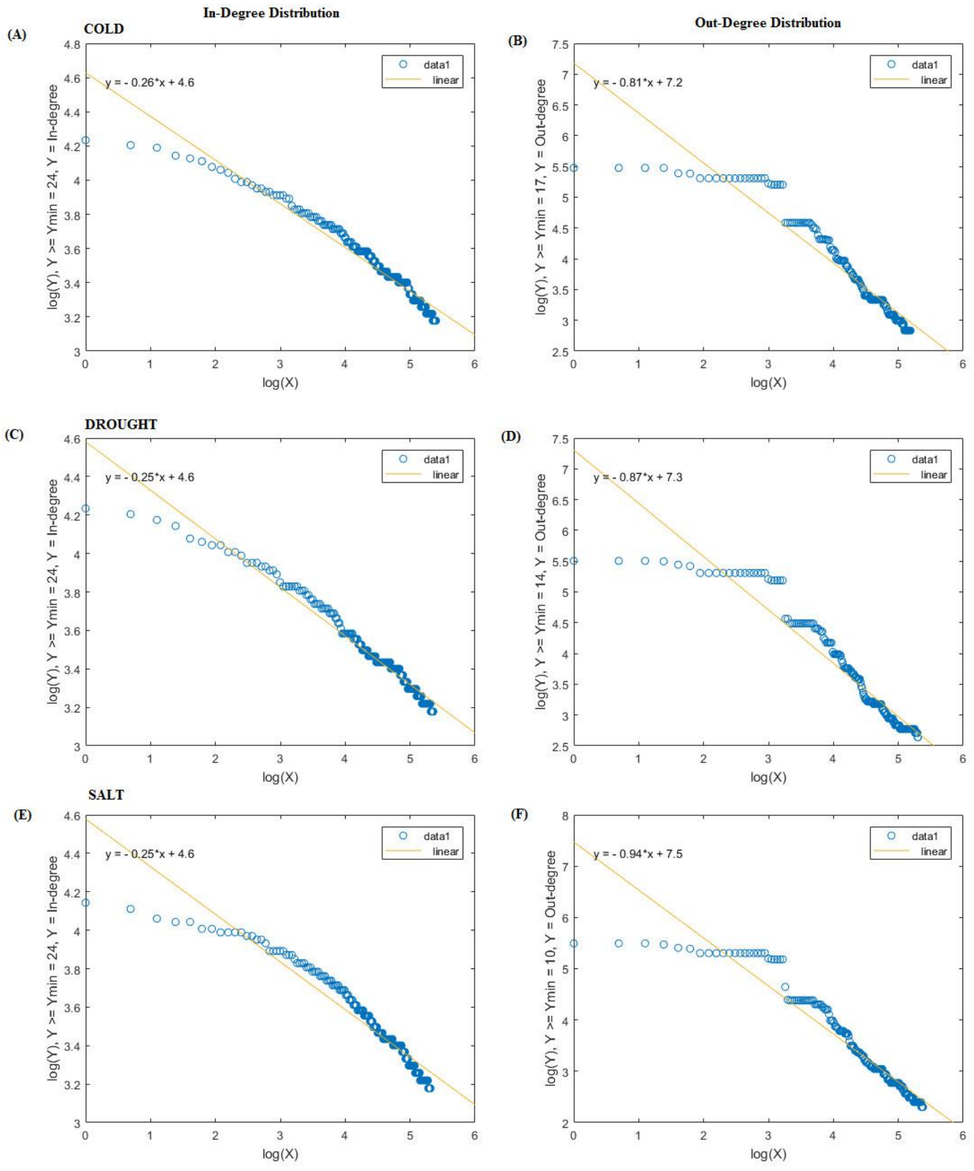

Figure S1: Degree distribution plot

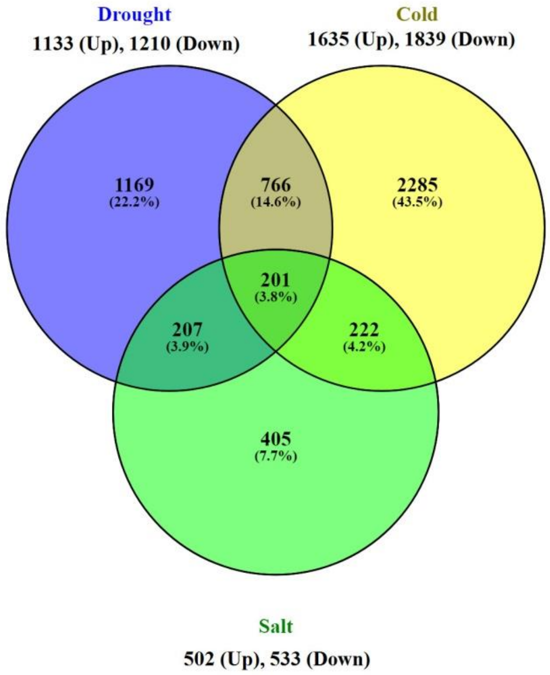

Figure S2A: Venn diagram for differentially expressed genes: Venn diagram depicting the number of up and down-regulated genes under drought, cold, and salt stress in *Oryza sativa*.

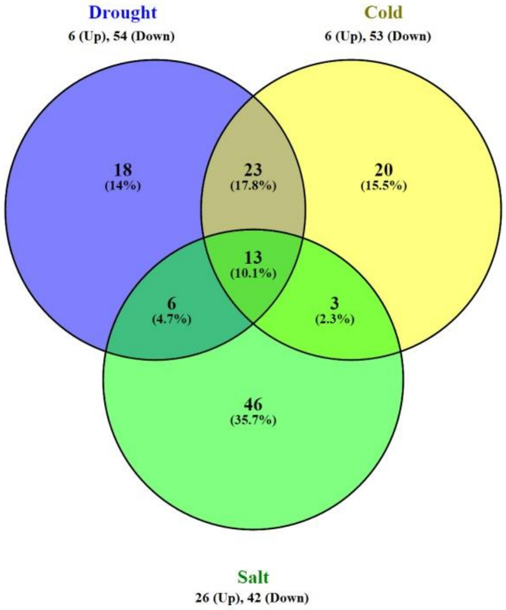

Figure S2B: Venn diagram for differentially expressed miRNAs: Venn diagram depicting the number of up and down-regulated miRNAs under drought, cold, and salt stress in *Oryza sativa*.

Supplementary Table Legends

Table S1: Details of transcriptome raw data
Table S2: List of differentially expressed genes under cold, drought, and salt stress in *Oryza sativa*
Table S3: miRNA Seq data analysis result
Table S4: List of differentially expressed miRNAs under cold, drought, and salt in *Oryza sativa*
Table S5: AGO1-enriched miRNA differentially expressed under abiotic stresses.
Table S6: List of miRNAs targets predicted by psRNATarget
Table S7: List of miRNAs targets identified by degradome sequencing data analysis
Table S8: List of miRNAs targets validated by degradome sequencing data analysis results
Table S9: Top ten genes/TF/miRNA ranked by degree, betweenness, and closeness centrality in *Oryza sativa* abiotic stress-induced regulatory networks
Table S10: Details of SCC found in three *Oryza sativa* abiotic stress-responsive miRNA-TF-gene regulatory networks

Table S11: Scale free network analysis
